# Agar Graft Modification with Acrylic and Methacrylic Acid for the Preparation of pH-Sensitive Nanogels for 5-Fluorouracil Delivery

**DOI:** 10.3390/gels10030165

**Published:** 2024-02-23

**Authors:** Ivelina Ivanova, Marta Slavkova, Teodora Popova, Borislav Tzankov, Denitsa Stefanova, Virginia Tzankova, Diana Tzankova, Ivanka Spassova, Daniela Kovacheva, Christina Voycheva

**Affiliations:** 1 Faculty of Pharmacy, Department Pharmaceutical Technology and Biopharmacy, Medical University—Sofia, 1000 Sofia, Bulgaria; ivelinavasileva23@gmail.com (I.I.); tpopova@pharmfac.mu-sofia.bg (T.P.); btzankov@pharmfac.mu-sofia.bg (B.T.); 2 Faculty of Pharmacy, Department of Pharmacology, Pharmacotherapy and Toxicology, Medical University—Sofia, 1000 Sofia, Bulgaria; denitsa.stefanova@pharmfac.mu-sofia.bg (D.S.); vtzankova@pharmfac.mu-sofia.bg (V.T.); 3 Faculty of Pharmacy, Department of Pharmaceutical Chemistry, Medical University—Sofia, 1000 Sofia, Bulgaria; d.tsankova@pharmfac.mu-sofia.bg; 4 Institute of General and Inorganic Chemistry, Bulgarian Academy of Sciences, 1113 Sofia, Bulgaria; ispasova@svr.igic.bas.bg (I.S.); didka@svr.igic.bas.bg (D.K.)

**Keywords:** pH-sensitive polymers, grafted agar, nanogels, 5-Fluorouracil, pH-sensitive delivery

## Abstract

Agar, a naturally occurring polysaccharide, has been modified by grafting it with acrylic (AcA) and methacrylic (McA) acid monomers, resulting in acrylic or methacrylic acid grafted polymer (AA-g-AcA or AA-g-McA) with pH-sensitive swelling behavior. Different ratios between agar, monomers, and initiator were applied. The synthesized grades of both new polymer series were characterized using FTIR spectroscopy, NMR, TGA, DSC, and XRD to ascertain the intended grafting. The percentage of grafting (% G), grafting efficiency (% GE), and % conversion (% C) were calculated, and models with optimal characteristics were further characterized. The swelling behavior of the newly synthesized polymers was studied over time and in solutions with different pH. These polymers were subsequently crosslinked with varying amounts of glutaraldehyde to obtain 5-fluorouracil-loaded nanogels. The optimal ratios of polymer, drug, and crosslinker resulted in nearly 80% loading efficiency. The performed physicochemical characterization (TEM and DLS) showed spherical nanogels with nanometer sizes (105.7–250 nm), negative zeta potentials, and narrow size distributions. According to FTIR analysis, 5-fluorouracil was physically incorporated. The swelling and release behavior of the prepared nanogels was pH-sensitive, favoring the delivery of the chemotherapeutic to tumor cells. The biocompatibility of the proposed nanocarrier was proven using an in vitro hemolysis assay.

## 1. Introduction

Nanotechnology is a rapidly developing and promising field in pharmaceutical science due to the versatile properties and applications it may provide over conventional drug delivery strategies [1]. The advantages of nanoparticles are their potential to pass through the smallest capillary vessels; avoidance of rapid clearance by phagocytes, thereby prolonging their stay in the bloodstream; penetration into cells and tissue to arrive at target organs; reduced toxicity of loaded drugs based on their controlled release [2]. Nanogels represent one of the many known nanocarriers. They are defined as nanoscale three-dimensional networks of hydrogel polymers with the ability to absorb significant amounts of water [3,4,5]. They simultaneously possess the properties of nanoparticles and hydrogels, which endows them with more advantages [6]. Their nanometer range can improve the enhanced permeation and retention effect (EPR) and improve the delivery of chemotherapeutics [5]. Based on the method of their preparation, nanogels can be classified chemically (covalently) or physically crosslinked [4]. The level of crosslinking can finely tune the drug release rate. The polymer can be obtained or polymerized from monomers together with the crosslinking step [7]. By utilizing newly synthesized polymers, new properties can be attributed to the nanogels, such as stimuli-sensitive delivery. The development of stimuli-sensitive multi-particulate formulation is a promising platform for sustained release and drug targeting [8,9]. The stimuli to which the used polymers respond—pH, temperature, light, magnetic field, enzymes, ionic strength, ultrasound, and redox change—depend on their properties [10]. One of the triggers used for smart delivery is the pH or proton concentration because of anatomical and pathological differences in the human body. It is well known that the gastrointestinal tract has pH-varying media. The stomach pH is between 1 and 3, in the duodenum 4.8–5.2, small intestines 6.8, and colon 7–7.5 [11]. In chemotherapy, the pH-dependent release is significantly exploited due to the difference between the physiological pH of 7.4 for the blood and normal cells and the neoplastic cells. The latter are characterized by lower extracellular pH (pH ≈ 4–5.8) [12,13] and 4.5–5.0 in the lysosomes [5]. Thus, the choice of polymer for the nanogel preparation can determine its properties. If the swelling/deswelling properties of the polymer are pH-dependent, then the drug release can be controlled for the desired place or cells [14]. Both natural and synthetic polymers can be used for the subsequent crosslinking. Some examples of natural polymers, such as agar, sodium alginate, chitosan, and dextran belong to the polysaccharide class. They are an object of biomedical scientific interest because of their biocompatibility, biodegradability, and enhanced adhesion with biological tissues such as epithelia and mucous membranes [15]. They all contain various functional groups, allowing grafting or crosslinking with synthetic polymers. This chemical modification could lead to stimuli-sensitive behavior [16]. Graft polymers are usually obtained through the use of initiators or gamma ray, UV, or microwave radiation in order to create a free radical site on the polymer chain. Monomeric units are attached similarly to a side chain to a polymer backbone to form a branched polymer with desirable properties non-intrinsic for the base polymer [17]. In regard to the pH-sensitive delivery, specific polymers which change their physicochemical properties based on the change in the pH can be utilized. They polyelectrolytes with ionizable groups whose solubility in aqueous of solutions is changeable by environmental pH. Typical monomers and polymers used for pH-sensitive systems include two types—anionic and cationic. Examples of anionic monomers are acrylic acid, methacrylic acid, propionic acid, ethylenesulfonic acid, styrenesulfonic acid, and cationic monomers include acrylamide, aminoethyl methacrylate, N,N′-dimethylaminomethylacrylamide, N,N′-dimethylaminoethyl methacrylate, N,N′-dimethylaminopropyl methacrylate, N,N′-diethylaminoethyl methacrylate, diallyldimethylammonium chlorid [18]. Acrylic and methacrylic acid have been employed to graft modification of agar for various purposes including waste water treatment, antimicrobial properties, or drug delivery [19,20,21,22]. Agar is a natural unbranched hydrophilic polysaccharide. One of its interesting properties is the capacity to form gels even at low concentration. It is biocompatible, inexpensive and can be easily chemically modified [23].

As a model drug in the current study, 5-Fluorouracil (5-FU) (5-fluoro-2,4-pyrimidinedione) was chosen. This drug is an antimetabolite used to treat colorectal, stomach, breast, pancreas, ovary, liver, and other solid tumors [24,25,26]. It is a pyrimidine analog and has good water solubility, mainly administered intravenously [26,27]. The major drawback of 5-FU clinical usage is the development of drug resistance. Moreover, the drug is associated with high toxicity. It can be manifested with several side effects, such as gastrointestinal, hematological, neural, dermatological, myelosuppression, and cardiotoxicity [28,29]. Its inclusion in different nanoparticles could affect its efficacy by increasing circulation time, reducing side effects, and improving its therapeutic index.

Even though the increasing use of nanoparticles in pharmaceutical technology provides opportunities for innovative approaches in therapy and diagnosis, the toxicity risk related to the new nanostructured drug delivery systems needs to be well established. The hemolytic assay serves as a primary screening tool for in vitro biocompatibility assessments of drugs and new drug delivery systems. Hemolysis refers to the breakdown of the erythrocyte membrane, leading to the release of hemoglobin into the plasma. Various factors contribute to hemolysis, such as immunologic responses, antigen–antibody reactions, mechanical injury, specific infections, hereditary and acquired cell membrane disorders, G6PD deficiency, hemoglobinopathies (e.g., sickle cell diseases, thalassemia), and certain chemotherapeutic agents [30]. This process causes anemia and poses a substantial limitation for the direct use of chemotherapeutic drugs [31].

The aim of the current study is to investigate the possibility of synthesizing agar-grafted pH-sensitive polymers with the help of acrylic or methacrylic acid and further utilize them as a polymer for nanogel preparation. The prepared nanogel could serve as a carrier for a model chemotherapeutic agent. Thus, it could be more efficiently and safely delivered to the target tumor cells where the pH-sensitivity of the nanogel could guarantee its release.

## 2. Results and Discussion

### 2.1. Preparation of Agar Agar-g-Polyacrylic Acid (AA-g-AcA) and Agar Agar-g-Polymethacrylic Acid (AA-g-McA) Polymers

Ag-g-AcA and AA-g-McA were synthesized by graft polymerization using cerium ammonium nitrate (CAN) as a free radical initiator in an inert nitrogen atmosphere. By direct oxidation, CAN generates free radicals on the agar backbone [17]. Graft copolymers are generated in the presence of these active free radicals and the presence of acrylic or methacrylic acid.

A series of polymers were synthesized, varying the ratio between agar, acid monomers, and the initiator. The investigated parameters, together with the sample coding, are presented in Table 1.

### 2.2. Characterization of Newly Synthesized Polymers

The effects of different ratios on the %G and %GE were investigated. Initially, with increasing monomer concentration, an increase in %G and %GE was observed, but after the optimal value was achieved, they decreased. The initial increase in grafting is probably due to the greater availability of grafting sites on the monomer. After that, the monomer remains in excess. With an excess of monomer, the formation of more homopolymer is observed compared with the grafted polymer. Additionally, upon homopolymer formation, the viscosity in the reaction medium increases, which creates an obstacle to the movement of free radicals to the active sites, resulting in lower %G and %GE.

The lower concentration of the initiator probably generates fewer free radicals to attack the AA backbone, resulting in lower values of %G and %GE. At the highest concentration, ceric ions increase their participation in the termination of growing grafted chains, and the parameters’ values decrease.

All obtained AA-g-AcA and AA-g-McA polymers show greater intrinsic viscosity values than agar at 35 °C. The obtained intrinsic viscosity of the agar was in agreement with previously determined values [32]. Therefore, the method could be used to calculate the viscosity of the average molecular weight. The increase in hydrodynamic volume measured by [η] correlates with higher molecular weight [33]. With the grafting of more AcA or McA units to the AA backbone, the $\bar{M\eta }$ of grafted polymer increases. After achieving the optimum ratio between polymer, monomer, and initiator the molecular weight decreases. These results are consistent with the literature [32].

Thus, the highest G% and GE% are obtained for A2 and M2 polymers, respectively (Table 1). These models have been further characterized. From now on, in the text, they will be referred to as AA-g-AcA and AA-g-McA for clarity.

**Figure 1:**
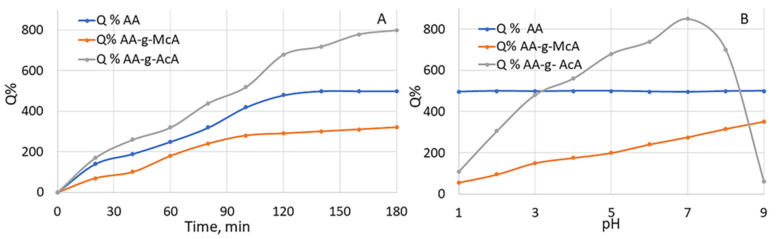
Effect: (**A**) of time on Q% (**B**) of pH on Q% of AA, AA-g-McA, AA-g-AcA.

The swelling behavior over time in deionized water and its dependence on pH were investigated for the two selected polymers. The results are depicted in Figure 1.

All of the tested polymers showed swelling upon contact with an aqueous medium (Figure 1A). This is expected because of their ability to absorb and retain water. The effect of pH on their swelling behavior is shown in Figure 1B. Q% values for AA are independent of pH because AA macromolecules do not contain weakly acidic or weakly basic groups. AA-g-AcA2 shows expressed pH-dependent swelling behavior. The presence of COOH groups in the weak polyacid and the increase in pH affect Q%. The ionization of carboxylate groups at pH values around 7 results in electrostatic repulsion and enhanced swelling. A further increase in the pH is associated with a screening effect of the present Na^+^ ions in the medium. Similar findings were reported for other polysaccharide hydrogels grafted with acrylic acid [34]. Dependence on pH is observed for the AA-g-McA polymer but to a lower extent. This is probably attributed to the presence of a hydrophobic methyl group in its structure as opposed to the AA-g-AcA polymer. Similar results of Q% about grafting with methacrylic acid were found in the literature [35,36,37]. Rapid deswelling in the case of AA-g-McA could be expected as well, but at higher pH values [38]. In the present study, the swelling behavior was investigated only in the physiologically relevant pH range, as the prepared hydrogel is intended for biomedical applications.

Moreover, the absolute value of Q% of AA-g-McA is lower than that of pure AA. That is probably due to the hydrophobic interactions between McA and AA, which further stabilize the structure of the resulting hydrogel after swelling. Therefore, the less stable AA-g-AcA swells to a greater extent at comparable pH values. In addition, the AA-g-AcA hydrogel loses its mechanical strength at pH = 8, which is expressed in forming a dispersion of insoluble particles.

Further, 1H NMR spectroscopy was applied to confirm the grafting of AA with acrylic and methacrylic acids using deuterated DMSO as solvent. Pure deuterated DMSO shows no peaks in 1H NMR spectroscopy and is commonly used as an NMR solvent. However, the commercially available samples are not 100% pure, and a residual DMSO-d5 1H NMR signal is observed at 2.50 ppm [39]. The 1H NMR spectrum of agar shows peaks at δ = G1′-5.22, A1-5.08 and agarose skeleton (G2–G6) and (A2′–A6′)-4.83–3.96 ppm. The 1H NMR spectrum of acrylic acid consists of three quadruplets centered at 6.52 ppm (cis proton), 6.14 ppm (germinal proton), and 5.96 ppm (trans proton) and proton of the carboxylic group at 12.0 ppm [40]. When the acrylic or methacrylic acid was grafted on AA, the peak at 3.96 ppm showed decreased signal strength as monomer moieties replaced the hydroxyl group. The AcA carboxylic group’s proton peak shifted to 12.16 ppm and 12.22 ppm for the McA carboxylic group.

Compared with the spectrum of AA, new resonances for AA-g-AcA and AA-g-McA new proton signals appeared at 1.5 ppm, 1.7 ppm, and 2.2 ppm for –OH and methylene protons of AcA, respectively, as well as 0.9 ppm and 1.7 and 2.2 ppm, because of the presence of –OH, methyl and methylene groups in McA respectively. These results demonstrated the formation of AA-g-AcA and AA-g-McA.

**Figure 2:**
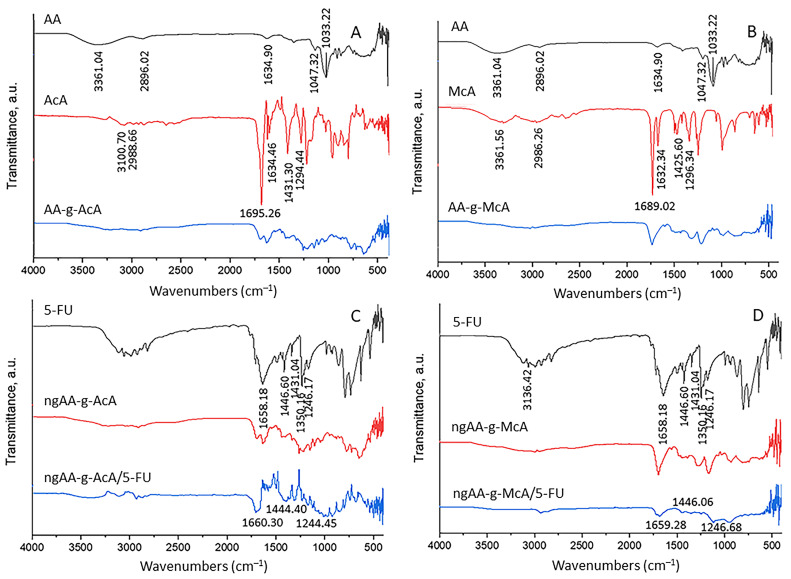
FTIR spectra of (**A**) AA (black line), AcA (red line), AA-g-AcA (blue line) and (**B**) AA (black line), McA (red line), AA-g-McA (blue line), (**C**) 5-Fluorouracil (black line), ngAA-g-AcA (red line), ngAA-g-AcA/5-FU, (**D**) 5-Fluorouracil (black line), ngAA-g-McA (red line), ngAA-g-McA/5-FU.

FTIR spectra of AA, AcA, McA, AA-g-AcA, and AA-g-McA were recorded to prove the successful grafting of AcA and McA. The results are presented in Figure 2A,B. The peak at 3361 cm^−1^ corresponds to stretching vibrations of hydroxyl groups on the spectrum of AA (Figure 2A,B). At 1033 cm^−1^, stretching vibrations of CH_2_OH are found in the same spectrum. The signal at 2896 cm^−1^ is related to C–H stretching vibration, and the one at 1634 cm^−1^ is associated with C=O stretching vibrations. The stretching for the C–O bond was observed at 1047 cm^−1^ [41]. Figure 2A,B show the FTIR spectrum of AcA and McA, respectively. The stretching vibration of –OH from the acid group is detected at 3100 cm^−1^, while at 1431 cm^−1^, the O–H bending vibration of the same group for AcA is at 3361 cm^−1^, and McA is at 1425 cm^−1^. The bending movement of terminal =CH_2_ is detected at 1294 cm^−1^ [42] for AcA and 1296 cm^−1^ for McA. At 2988 cm^−1^, the C–H stretching of the allyl group for AcA and at 2986 cm^−1^ for McA. The carbonyl group is discovered at 1695 cm ^−1^ for AcA and 1689 cm^−1^ for McA, and the vinyl group’s stretching vibration signal at 1634 cm^−1^ for AcA and 1632 cm^−1^ for McA guarantees the absence of homopolymerization for both monomers. McA shows a peak at 1375 cm^−1^ due to CH_3_ bending.

**Figure 3:**
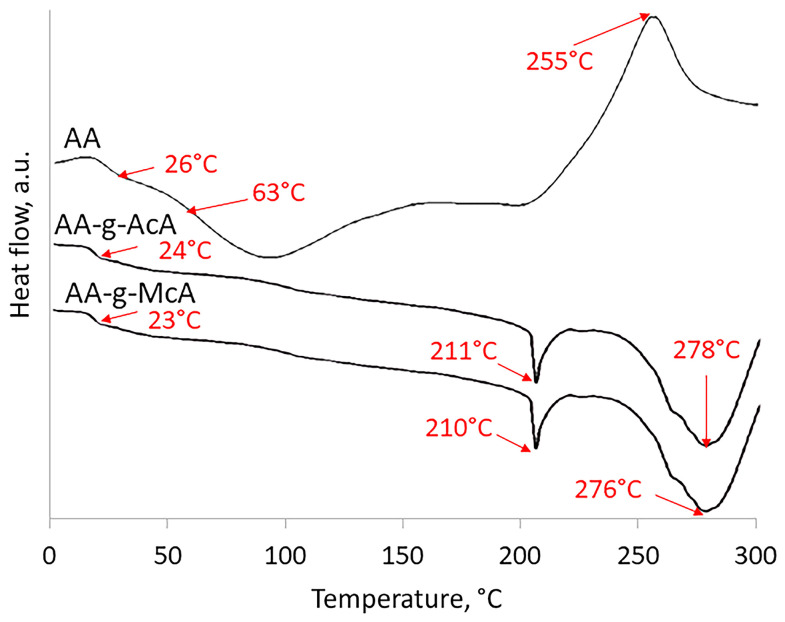
DSC profiles of AA, AA-g-AcA, and AA-g-McA.

The difference between the monomer and the grafted polymer spectra is the absence of a signal at 1634 cm^−1^ for the vinyl group of AcA and a signal at 1632 cm^−1^ for McA. In addition, the absence of the stretching vibrations found in the AA spectrum of CH_2_OH at 1033 cm^−1^ in spectra of grafted polymers shows the successful grafting of monomer units onto the AA backbone. The thermal behavior of AA, AA-g-AcA, and AA-gMcA were compared using DSC analysis at a heating rate of 10 °C/min to distinguish thermal transitions. Agar composition exhibited two glass transitions (Tg). One was observed at 26 °C by agarose and another at 63 °C by agaropectin. The exothermal signal noticed at 255 °C is associated with its thermal decomposition (Figure 3). AA-g-AcA shows Tg at 24 °C, as well as two endothermic peaks at 211 °C and the other at 278 °C while AA-g-McA shows Tg at 23 °C and endothermic peaks at 210 °C and the other at 276 °C. The signals noticed at 23 and 24 °C are Tg belonging to AA on the grafted polymer. The 211 °C and 210 °C peaks were new and corresponded to the AA-g-AcA and AA-g-McA melting points. They are assigned by the formation of the new polymeric structure by the combination of AA and AcA or McA. The grafted polymers’ thermal profiles demonstrated the presence of agar on their structure and the obtention of new polymeric material. Additionally, the AA-g-AcA and AA-g-McA exhibited an enhancement in thermal resistance compared with agar since no exothermic signals were observed.

**Figure 4:**
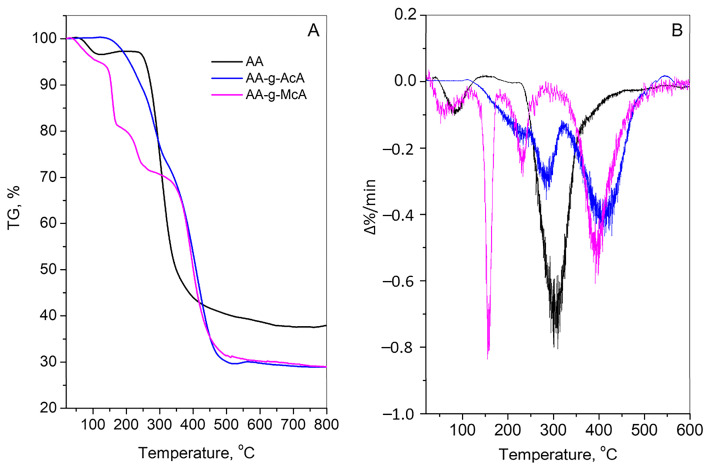
TGA (**A**) and DTG (**B**) curves of initial AA and the grafted polymers AA-g-AcA (A4) and AA-g-McA (M4) in Ar.

Additionally, the thermal stability of the initial AA and the grafted polymers was studied using thermogravimetric analysis. Figure 4 shows the TG and DTG curves for the thermal decomposition of AA and the grafted polymers AA-g-AcA and AA-g-McA in Ar. The TG curve for AA comprises two thermal steps: first small one up to ~120 °C, due to loss of physically absorbed water, which is about 3% of the mass, and second large step at 250–350 °C with a mass loss of ~50%, due to the degradation of agar [43,44]. The DTG curve of AA revealed the main decomposition peaks are centered at 85 °C and 300 °C.

The final mass loss is 62% up to 800 °C. At the thermogram of AA-g-AcA, a gradual mass loss (22%) up to about 300 °C due to the loss of free water and dehydration of the polymer backbone is observed. The sharp step registered from 300 to 500 °C with a mass loss of 50% should be attributed to de-polymerization and degradation of the AA and AcA compounds. The DTG maxima are found to be ~290 °C and ~10 °C. The final mass loss is about 71% up to 800 °C. Four well-established thermal stages are visible in the AA-g-McA TG curve. The first one resembles those of AA with a mass loss of 5% up to 125 °C due to loss of physically absorbed water. The second stage at temperatures 130–200 °C is usually interpreted as water release due to a reaction between carboxylic groups of the McA forming polymethacrylic anhydride [45,46]. The next step, with 9% mass loss at 200–300 °C, could be due to de-polymerization, and the final stage, 310–520 °C is associated with the degradation of the residual fragments to form carbonaceous material [46,47]. The corresponding maxima of DTG peaks of degradation are registered at 75 °C, 160 °C, 230 °C and 400 °C. In the case of AA-g-McA, the final mass loss is 71%. Thermal behavior data for the samples, according to a study [48], are placed in Table 2.

**Figure 5:**
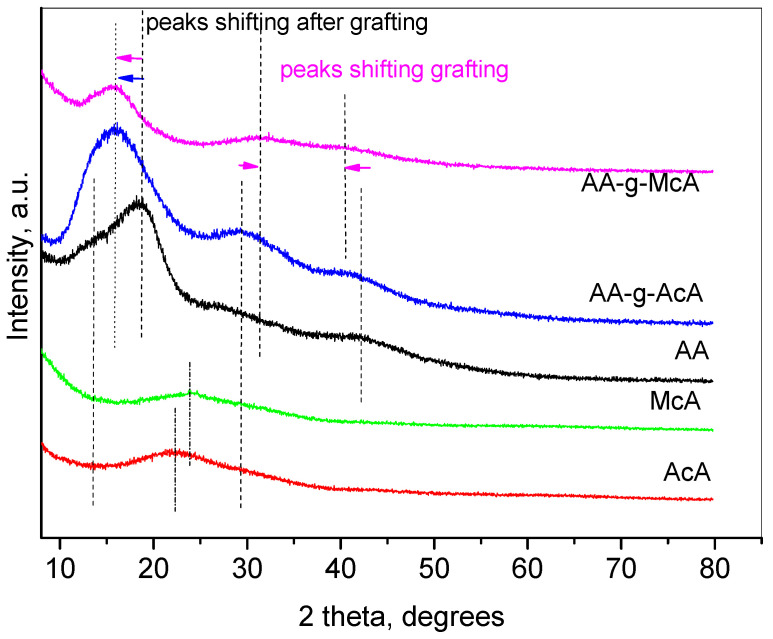
X-ray powder diffraction patterns of the initial compounds and the grafted polymers.

X-ray powder diffraction was also applied for the polymer characterization. Figure 5 represents X-ray powder diffraction patterns (PXRD) of initial compounds—agar (AA), Acrylic acid (AcA), and Methacrylic acid (McA) as well as that of the prepared grafted polymers—(AA-g-AcA) and (AA-g-McA). The PXRD pattern of agar (AA) consists of four amorphous peaks. The first one is at 13.5°2θ and appears as a shoulder of the second peak at 18.6°2θ, which is the most intensive peak of the diffractogram. The intensity ratio between these two peaks is a measure of the degree of hydration and crystallinity of the agar structure [49]. Two more peaks with lower intensities are visible at 29.9°2θ and 42.7°2θ. The patterns of acrylic and methacrylic acids are similar and consist of broad humps at about 22.6°2θ and 24.1°2θ, respectively.

The patterns of the grafted polymers AA-g-AcA and AA-g-McA are also similar, showing three more or less intensive peaks. The transformation of the aar peaks from a clear doublet to a single peak in the range of 13–18° 2θ after the interaction with the acrylic and methacrylic acids indicates the successful formation of the grafted polymers. For AA-g-AcA, the first one is at 15.9° 2θ, the next peaks are at 29.6° 2θ and 41.1° 2θ while for AA-g-McA, the first one appears at 15.9° 2θ also but the second and third peaks could be found at 31.2° 2θ and 40.6° 2θ. Compared with the pure agar pattern, the shift of the three peaks appears more pronounced for the sample with methacrylic acid, which may be an indication that the latter modifies the chain structure of agar to a higher extent [38].

### 2.3. Preparation of Nanogels—Parent and 5-FU Loaded

The newly prepared graft polymers were subsequently applied in the preparation of nanogels by crosslinking with glutaraldehyde (GA). The preparation followed an established protocol with modifications [50]. GA is the crosslinker most frequently used to prepare polymeric hydrogel beads, microspheres, and nanogels [51]. The ratio between polymer, monomer, and crosslinker, as well as drug loading efficiency and swelling, were investigated, and the results are summarized in Table 3. The resulting nanogels were named as follows: parent nanogels AA-g-AcA (ngAA-g-AcA), AA-g-McA (ngAA-g-McA), loaded nanogels AA-g-McA (ngAA-g-McA/5-FU), and AA-g-McA (ngAA-g-McA/5-FU).

### 2.4. Characterization of Nanogels

Nanogels were prepared in different drug/polymer ratios and crosslinker amounts, and these data are presented in Table 3. Increasing the amount of polymer in the drug/polymer ratio results in the retention of a greater amount of 5-FU in the nanogels. It is most likely due to increased hydrogen bonds that the drug and the polymer create between themselves because of the larger number of hydroxyl groups on the polymer’s side. The drug loading efficiency decreases with an increase in the amount of drug used during the preparation of the nanoparticles. Maximum encapsulation efficiency (EE%) was found at a drug/polymer ratio of 1/3 for both polymers. The amount of 5-FU above the maximum encapsulation efficiency may be washed after the lavation of the obtained particles because of the drug’s good water solubility. At the same time, increasing the amount of GA leads to an increase in the EE% value for both polymers. It is most likely due to the tighter crosslinking, which allows a greater amount of 5-FU to be retained. Based on the results of the drug loading efficiency calculations, the experiments continued with the nanoparticles prepared with a drug/polymer ratio of 1/3 and a 4 mL crosslinker.

FTIR spectra of 5-FU, ngAA-g-AcA, ngAA-g-McA, ngAA-g-AcA/5-FU, and ngAA-g-McA/5-FU were recorded to prove the loading of 5-FU. The spectra can be seen on Figure 2C,D. The spectrum of 5-FU shows characteristic picks at 3136 cm^−1^ due to N-H stretching, C=O stretching at 1658 cm^−1^, C=C stretching at 1446 cm^−1^, C-F stretching at 1431 cm^−1^, C-N stretching at 1246 cm^−1^, and vibration of the pyrimidine ring at 1350 cm^−1^ [52,53].

It was observed that there were no changes in these main peaks in the IR spectra of ngAA-g-AcA/5-FU and ngAA-g-McA/5-FU, which assumes the physical incorporation of 5-FU in both types of nanoparticles.

**Figure 6:**
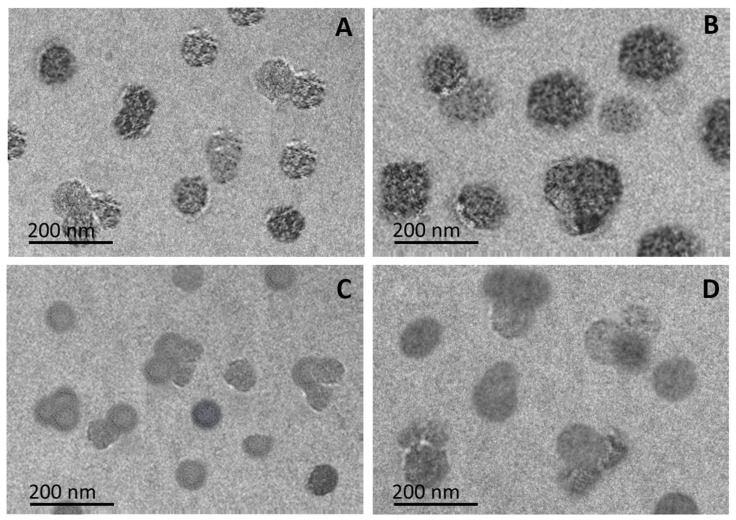
Transmission electron micrographs of empty ngAA-g-AcA (**A**), 5-FU loaded ngAA-g-AcA (**B**), empty ngAA-g-McA (**C**), and 5-FU loaded ngAA-g-McA (**D**) nanoparticles.

Further, the size, shape, and structure of ngAA-g-AcA and ngAA-g-AcA/5-FU as well as ngAA-g-McA and ngAA-g-McA/5-FU were studied using TEM (Figure 6). As seen, empty nanoparticles show spherical shape, nanoscale size, matrix structure, and narrow distribution. The same shape, structure, and distribution are observed for the loaded nanoparticles. The increase in size after loading is visible.

The obtained TEM data are in accordance with the DLS analysis. The particle sizes are in the nano range for the obtained nanogels (Table 4). The nanogels’ diameter for both polymers increased with the loading of the drug. Probably, the entrapment of the drug in the free volume areas of the polymer particles prevents the additional shrinking. The AA-g-McA nanogels show a smaller size compared with the AA-g-AcA. Most likely, the higher viscosity of the polymer with attached AcA units forms larger droplets when the polymer solution is sprayed into the crosslinker solution, resulting in a larger particle size. This fact is also confirmed by the results obtained from the swelling test. ngAA-g-AcA swells to a greater extent compared with ngAA-g-McA (Table 4). Similar results regarding the nanogels’ swelling behavior have been reported before [54].

The PDI value shows the physical stability of the empty nanogels and narrow limits in size distribution. Upon 5-FU loading, the polydispersity index for both polymer-based nanogels decreases, most probably due to a change in the zeta potential value. The 5-FU molecule (pKa = 8.0) is negatively charged in distilled water [55]. After loading, empty polymer particles accumulate the drug’s negative charge, reflecting on the zeta potential value. This hypothesis is supported by FTIR data showing the physical entrapment of the drug within the nanogels.

**Figure 7:**
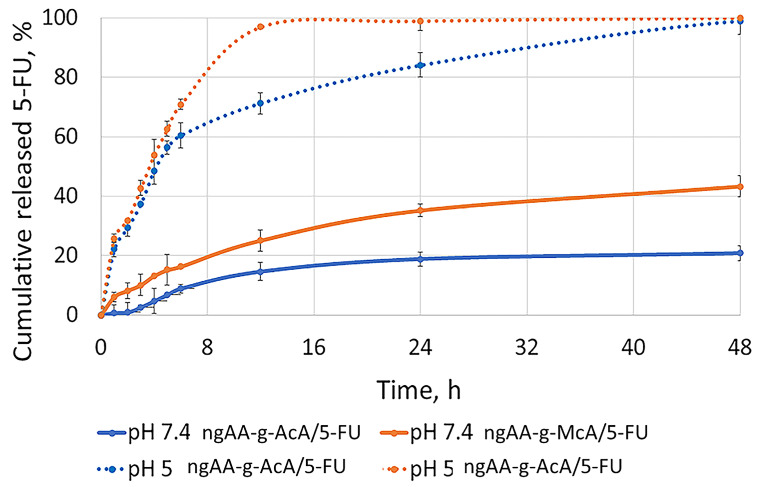
Release profiles of 5-Fluorouracil from npAA-g-AcA and npAA-g-AcA in a buffer medium with a pH of 7.4 and pH of 5.0 at temperature 37 °C, 100 rpm; n = 3 ± SD.

The pH-dependent release of 5-FU was observed by performing an in vitro dissolution test in pH 7.4 and 5.0 buffer media at 37 °C. The results are presented in Figure 7.

At pH 7.4, the nanogels obtained from both polymers showed a sustained release. For ngAA-g-AcA, it was about 20% in 48 h, and for ngAA-g-McA, it was about 50%. This delay is most likely due to the swelling of the polymer under these conditions. Swelling significantly slows the release of the drug due to the additional crosslinking of the polymer that occurs during the process of preparation of the nanoparticles. The obtained gel layer, by the swelling of the polymer, slows down the release of 5-FU.

At pH 5.0 (Figure 7), ngAA-g-AcA/5-FU and ngAA-g-McA/5-FU show increased and faster release. Such pH conditions are associated with the tumor cells because of their extensive metabolism [16]. The nanogels at the simulated tumor conditions (pH 5.0) show an initial burst release for the first 6 h. A gradual, controlled release was observed over time, and almost 100% of the released 5-FU from ngAA-g-McA was reached at 12 h and 100% from ngAA-g-AcA at 24 h. The faster release is probably connected with polymers’ swelling behavior, which is used for nanogel preparation and physical entrapment of 5-FU in them. At pH 5.0, both polymers swell to a lower extent, which allows faster drug release.

The slower release of 5-FU at physiological pH (7.4) is considered to be an advantage as it may reduce drug loss until it reaches the target tumor tissue and is a prerequisite for lowering systemic toxicity. In the bloodstream, as the particles pass from a pH 7.4 region to a pH 5.0 region, the release would be promoted due to the contraction of the polymer chains and the release of API located in the voids of the swollen polymer at pH 7.4. This is in accordance with equilibrium swelling data presented in Table 3. Similar results have been reported in other studies [56].

**Figure 8:**
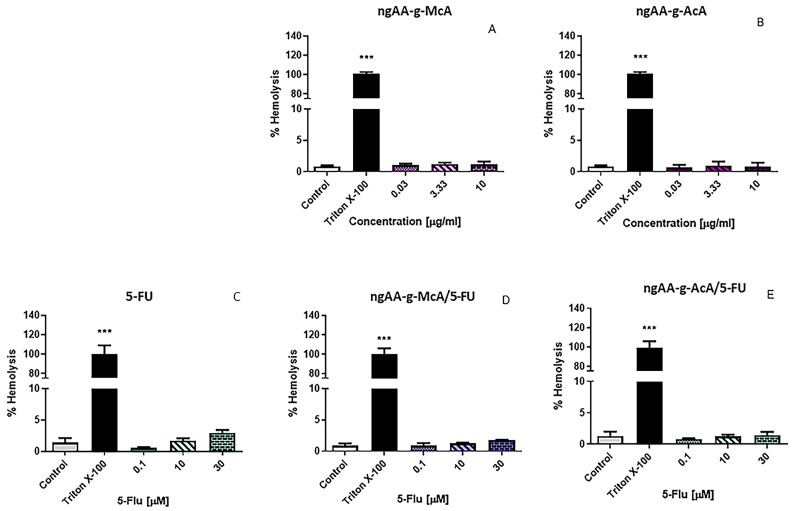
Hemolytic effects of (**A**) AA-g-McA, (**B**) AA-g-AcA, (**C**) free 5-FU, (**D**) 5-FU loaded in AA-g-McA, (**E**) 5-FU loaded in AA-g-AcA on human erythrocytes. Data are presented as means ± SD from triplicate assays (n = 3). Statistical comparisons were made against the untreated controls using one-way ANOVA followed by Dunnett’s post-test *** *p* < 0.001 vs. control.

Nanosized drug delivery systems are designed for different routes of administration (e.g., oral or parentheral use), but in most cases, these systems would have direct contact with circulating blood. The hemocompatibility of nanoparticulate systems, as a part of their preliminary biocompatibility evaluation, includes an assessment of their hemolytic activity as an important safety marker. In this study, the hemolytic activity of the tested compounds was compared with Triton X-100 (20%) in erythrocyte samples (see Figure 8). After 1 h incubation, Triton X-100 led to complete hemolysis (100%). The empty nanogels (ngAA-g-McA and ngAA-g-AcA) exhibited no hemolytic effects (Figure 8A,B). Free 5-FU caused weak hemolysis (2.94%) at the highest concentration (30 µM) (Figure 8C). At the same concentration (30 µM), 5-FU loaded in ngAA-g-McA/5-FU and ngAA-g-AcA/5-FU nanoparticles induced lower levels of hemolysis (1.45% and 1.73%, respectively), compared with the free drug, which is however below the threshold level of 5% according to ISO 10993-4 [57]. Our results are in accordance with the findings of Belman-Flores et al. (2020), who found a good hemocompatibility of pH-sensitive hydrogel nanoparticles based on N-isopropyl acrylamide (NIPAM) and methacrylic acid (MAA) [58]. In another interesting study performed by Zhang et al. (2019), the membrane integrity of poly(N-isopropylacrylamide) (PNIPAM)—treated red blood cells showed that hemolysis was lower than 1%, thus proving that PNIPAM did not significantly impaired red blood cells membrane integrity at a concentration of up to 10 mg/mL [59]. Moreover, data from our study support data from other studies indicating that incorporating 5-FU in different drug delivery systems could be a successful approach to reducing its hemolytic potential [31,60].

## 3. Conclusions

In the present study, the grafting of acrylic or methacrylic acid onto agar was successfully carried out by free radical polymerization using cerium ammonium nitrate as an initiator. The optimal polymers were further utilized to prepare 5-FU loaded nanogels by crosslinking with glutaraldehyde. The results demonstrated that the drug release depends on the pH of the medium, being about 40% and 20% for ngAA-g-AcA/5-FU and ngAA-g-McA/5-FU, respectively, in pH 5.0. On the other hand, at pH 7.4, the 5-FU release is more pronounced and complete within 8 h from ngAA-g-McA/5-FU and within 48 h from ngAA-g-AcA/5-FU. Therefore, it can be expected that ngAA-g-AcA/-FU could provide release of the chemotherapeutic predominantly at the target site over a prolonged period of time. Data from biocompatibility characterization showed that both synthesized nanogels were hemocompatible with red blood cells; moreover, the loading of 5-FU in ngAA-g-McA and ngAA-g-AcA leads to a decrease in the hemolitic potential of the chemotherapeutic drug.

## 4. Materials and Methods

### 4.1. Materials

Agar-agar powder (AA), Acrylic acid (AcA), Methacrylic acid (McA), Ammonium ceric nitrate (CAN), Nitric acid, and glutaraldehyde were purchased from Sigma-Aldrich (St. Louis, MO, USA). Hydrochloric acid, Potassium dihydrogen phosphate, and Disodiumhydrogen phosphate dihydrate were purchased from Merck (Darmstadt, Germany). Hydroquinone was purchased from TCI Europe (Zwijndrecht, Belgium). Deionized water was prepared in the laboratory.

### 4.2. Methods

#### 4.2.1. The Preparation of Agar Agar-g-Polyacrylic Acid (AA-g-AcA) and Agar Agar-g-Polymethacrylic Acid (AA-g-McA)

**Figure 9:**
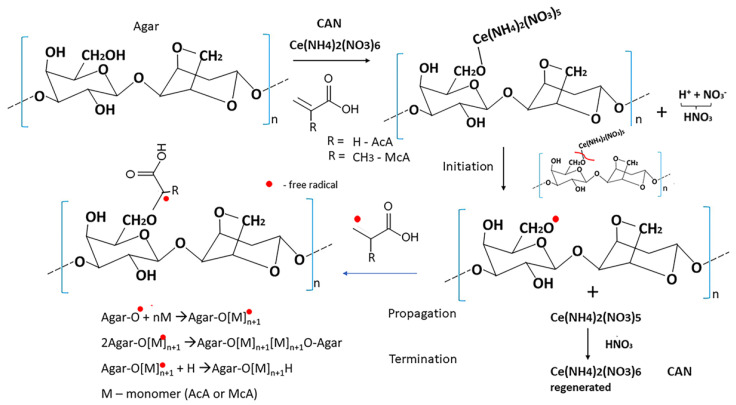
Schematic representation of mechanism for preparation of Ag-g-AcA and AA-g-McA in an inert atmosphere.4.2.2. Preparation of nanogels—parent and 5-FU loaded.

Agar was dissolved in distilled water at 90 °C in a three-neck round bottom flask with a gas inlet system and a condenser. Then, acrylic or methacrylic acid was added to the agar solution and stirred. Nitrogen gas was poured for 60 min before the solution of CAN in water, acidified with concentrated HNO_3_, was added, and the flask was closed. The ratio between polymer, monomer, and initiator was varied, and it is shown in Table 1. Then, the reaction was continued under constant stirring in a thermostatic paraffin bath for 6 h at a constant temperature of 40 °C. Finally, the grafting procedure was terminated upon adding a saturated hydroquinone solution. Next, a separating funnel splits the resulting polymer AA-g-AcA (A1-5) or AA-g-McA (M1-5) from the homopolymer. Next, the graft copolymer hydrogel was precipitated over acetone, separated by centrifugation, dried to constant weight, and ground for further use. The proposed synthesis mechanism of AA-g-AcA and AA-g-McA is represented in Figure 9.

The nanogels ngAA-g-AcA and ngAA-g-McA parent containing 5-FU were prepared as per a modified protocol [50]. Briefly, the polymer is placed in distilled water or a solution of 5-FU (0.1 mg/20 mL) and stirred to form a homogenous solution for 2 h. Then, it was added by spraying into stirred water containing glutaraldehyde (crosslinker) and HNO_3_ (catalyst) using a spraying device under ultrasound for 4 min (0.04 wats). The formed nanogels were then removed from the crosslinking solution by centrifugation and washed with water repeatedly to remove the glutaraldehyde and acid residue. Due to the known toxic effect of GA, its total amount was kept as low as possible during the crosslinking procedure, and the free GA was further eliminated. Finally, the nanogels were completely dried under vacuum at 40 °C. The varying ratios between polymer, monomer, and crosslinker are shown in Table 3. The resulting nanogels were named as follows: parent nanogels AA-g-AcA (ngAA-g-AcA), AA-g-McA (ngAA-g-McA), loaded nanoparticles AA-g-McA (ngAA-g-McA/5-FU), and AA-g-McA (ngAA-g-McA/5-FU).

#### 4.2.2. The Percentage of Grafting (% G) and Grafting Efficiency (% GE) were Calculated Using the Following Formulas

(1) $$\%Grafting=\frac{W_{2}-W}{W}\times 100$$

(2) $$\%\mathrm{Grafting}\;\mathrm{efficiency}=(\frac{W_{2}-W}{W_{1}})\;\times \;100$$

where W is the weight of AA, W_1_ is the weight of AcA or McA, and W_2_ is the weight of the grafted polymer.

#### 4.2.3. Transform Infrared Spectroscopy (FTIR)

AA, AcA, McA, AA-g-AcA, and AA-g-McA, as well as 5-FU, ngAA-g-AcA, ngAA-g-McA, ngAA-g-McA/5-FU, and ngAA-g-McA/5-FU were characterized using FTIR-ATR spectroscopy with a Thermo-Nicolet FTIR instrument (Thermo Fischer Scientific, Waltham, MA, USA) in the range of 4000–400 cm^−1^ and with a resolution of 4 cm^−1^.

#### 4.2.4. Nuclear Magnetic Resonance (NMR)

A Bruker AV600 spectrometer (Bruker AV600, Berlin, Germany) was used to acquire 1H-NMR at 250 MHz. The 1H–NMR spectra were measured with solutions of approximately 0.03 M. DMSO-d6 was used as a solvent. As an internal standard, chemical shifts were expressed as δ values in parts per million (ppm) against tetramethylsilane (TMS).

#### 4.2.5. Differential Scanning Calorimetry (DSC)

DSC curves of pure AA and graft copolymers AA-g-AcA and AA-g-McA were recorded using a differential scanning calorimeter PerkinElmer DSC-8500 (Waltham, MA, USA), equipped with an Intracooler 3 cooler. The samples were loaded into standard aluminum pans and were then scanned. The temperature range was from −50 °C to 180°C with a heat rate of 10 °C/min and from 180 °C to 20 °C with a cooling rate of 20 °C/min. The control of the device, data collection, and processing were performed with the help of specialized Pyris software v.10.1.0.0412.

#### 4.2.6. Thermogravimetric Analysis (TGA)

Thermogravimetric measurements were performed by LABSYSEvo and SETARAM (Caluire, France) in an argon flow within a temperature range of 20–800 °C and a heating rate of 10 °C/min.

#### 4.2.7. X-ray Diffraction Analysis (XRD)

Powder X-ray diffraction was performed on a Bruker D8 Advance diffractometer (Bruker AXS GmbH, Karlsruhe, Germany) with Cu K_α_ tube (λ = 0.15418 nm) equipped with LynxEye detector, with steps of 0.02°2θ in the region 5–80°2θ.

#### 4.2.8. Viscosity Measurements

The specific viscosities of AA, AA-g-AcA, and AA-g-McA were determined with an Ubbelodhe viscometer (Laborxing, Shenzhen, China) at 35 °C according to the procedure suggested by Rochas and Lachaye [33] using the formula:

(3) $$\eta_{sp}=\frac{t-t_{0}}{t_{0}}$$

where the specific viscosity is η_sp_, t, and t_0_ are the flow time in seconds of the polymer solution and pure solvent through the Ubbelohde viscometer.

Aqueous solutions of agar, AA-g-AcA, and AA-g-McA (0.005–0.05% *w*/*v*) were prepared using 0.75 M NaSCN to inhibit Agar aggregation.

Using η_sp,_ the reduced viscosity was calculated using the following formula:

(4) $$\eta_{red}=\frac{\eta_{sp}}{C}$$

where C is concentration in g/mL.

Extrapolating the reduced viscosity versus C (η_sp_/C) at 0 concentration, the intrinsic viscosity was obtained.

The viscosity average molecular weight ($\bar{M\eta }$) was determined using the relation with intrinsic viscosity given by the Mark–Houwink–Sakurada equation:

(5) $$\mathrm{Intrinsic}\;\mathrm{viscosity}\;[\eta ]=K(\bar{M\eta })$$

Based on literature data [33], the values of parameters α and K, depending on the polymer-solvent system, were taken assuming that they did not change with the grafting (K = 0.07mL/g, α = 0.72).

#### 4.2.9. Determination of Swelling Indices Q at Different pH

The grafted polymers’ swelling behavior (pH-dependent) was studied at varying pH (1–9) and at 20 min time intervals from 20 to 180 min in deionized water, and swelling indices (Q%) were calculated.

Dried samples of the grafted polymer (10 mg) were placed in a previously weighed (M) tube, and 20 mL water or a series of buffer solutions with pH values ranging from 1 to 9 were added. At equal time intervals, the liquid was removed through suction, and the sample tube was then weighed to determine the quantity of water absorbed per mg of grafted polymer. The experiment continued until a constant mass was achieved (M_s_), and the polymer swelled. The following formula is used to calculate the Q% value:

(6) $$Q\%=\frac{M_{s}-M}{M}\times 100$$

#### 4.2.10. Determination of Swelling Behavior of the Nanoparticles

The equilibrium swelling behavior of AA-g-AcA and AA-g-McA nanogels were studied in buffer solutions with pH 5.0 and pH 7.4 at 37 °C by gravimetric analysis following a reported procedure with some modifications [54]. A sample of the nanogels was placed in a previously measured cellulose membrane bag. It was placed in 25 mL of the corresponding buffer solution until complete equilibration. Afterward, the excess liquid was removed, and the swollen nanoparticles were weighed. The percent Equilibrium swelling degree (ESD) was calculated as follows.

(7) $$\mathrm{ESD}\;\%=\frac{M_{s}-M_{d}}{M_{d}}\times 100$$

where M_s_ is the mass of the nanogels in the swollen state, and M_d_ is the mass of the dry nanogels.

#### 4.2.11. Transmission Electron Micrograph (TEM) Characterization

The size and structure of the samples were characterized using transmission electron microscopy (JEOL JEM 2100 h STEM (200 kV; point-resolution 0.23 nm) JEOL (Freising, Germany) Samples were prepared by placing a water suspension of the nanoparticles on a polymer microgrid supported on a Cu grid.

#### 4.2.12. Dynamic Light Scattering (DLS)

The nanogel size, polydispersity index, and zeta potential were determined using a Zetasizer (Zetasizer Nano ZS, Malvern Instruments, Worcestershire, UK). Immediately after purification and before vacuum drying, the aqueous dispersion samples were measured at a scattering angle of 90° and 25 °C.

#### 4.2.13. Drug Loading Efficiency and Release

The 5-FU content of nanogels was estimated in deionized water using the extraction method [61]. The desired amount of 5-FU-loaded dry nanoparticles was extracted in 100 mL of deionized water at room temperature under stirring until all the drug was removed from the nanogels. After that, the polymer suspension was removed by passing it through a filter paper, and the filtrate was collected. The 5-FU content in the filtrate was measured with a UV spectrophotometer (Thermo Scientific Evolution 300, Madison, WI, USA) at a wavelength of 270 nm using the calibration curve of the series of 5-FU solutions with standard concentrations. The percent of entrapment efficiency was calculated according to the following equation.

(8) $$\mathrm{Encapsulation}\;\mathrm{Efficiency}\;\%\;(\mathrm{EE}\%)=\frac{5-FU\;total-5-FU\;filtrate}{5-FU\;total}\times \;100$$

The in vitro drug release from the nanoparticles was studied in a phosphate buffer pH 7.4 and 5.0, the physiological pH of blood serum, and the intercellular pH of neoplastic cells, respectively. The freshly prepared nanogel dispersion was introduced into a dialysis membrane bag (MW = 6000–8000 kDa), placed into 40 mL of release medium, and incubated in a shaking water bath at 37 °C with a speed of 100 rpm. At appropriate time intervals, an aliquot of the samples was withdrawn, and the amount of 5-FU released from the nanogels was evaluated using a UV spectrophotometer at a λmax of 270 nm. Then, an equal volume of fresh dissolution medium was added back to maintain a constant volume. All the experiments were performed in triplicate, and the presented results show the mean value.

#### 4.2.14. Hemolysis Assay

The test substances were assessed for their hemolytic potential using the methodology outlined in Evans et al.’s 2013 protocol [62]. Blood specimens from healthy volunteers were acquired from a certified clinical laboratory (Bodimed, Sofia, Bulgaria). All experimental procedures were performed in accordance with the rules of the Institutional Ethics Committee (KENIMUS) at the Medical University—Sofia, Sofia, Bulgaria [63]. Erythrocytes were isolated from blood through successive centrifugation in 0.9% NaCl buffer. The blood cells underwent resuspension in phosphate buffer (pH 7.4). In 96-well plates, test substances (empty AA-g-McA (0.03–10 mg/mL), empty AA-g-AcA (0.03–10 mg/mL); 5-FU (0.1–30 µM) and 5-FU in AA-g-McA, and 5-FU in AA-g-AcA, loaded in corresponding equimolar concentrations), along with 20% Triton X-100 (utilized as a positive control), and phosphate buffer (utilized as a negative control) were dispensed. Then, the erythrocyte suspension in phosphate buffer was introduced to these plates. Incubation at 37 °C was performed for 1 h, followed by centrifugation at 500× *g* for 5 min. The resulting supernatant was transferred to new 96-well plates, and the hemoglobin absorbance was measured at 430 nm using a Synergy 2 plate reader (BioTek Instruments, Inc, Highland Park, Winooski, VT, USA). The outcomes were expressed as the percentage of hemolysis concerning the hemoglobin absorbance values in the positive controls, with the hemoglobin absorbance of negative controls regarded as zero hemolysis. Substances causing hemolysis below 5% (in accordance with the acceptable hemolytic threshold defined by ISO 10993-4) were considered biocompatible [57] 

#### 4.2.15. Statistical Analysis

One-way ANOVA followed by a Dunnett post-hoc test was employed for statistical analysis. On GraphPad Prism software (version 8, Informer Technologies, Inc., CA, USA). The significance was determined at *p* < 0.001. These data are presented as mean ± SD (n = 3) derived from three independent experiments.

## Figures and Tables

**Table 1:** Polymer, monomer, and initiator ratios for the model grafted polymers. Characteristics of the grafted polymers $(\bar{M\eta })-$ viscosity average molecular weight; G%—grafting; GE%—grafting efficiency (see Equations (1) and (2)); η_intr_ 35 °C—intrinsic viscosity at 35 °C).

Code	Wt. of Agar (g)	Wt. of AcA (g)	Wt of McA (g)	Wt of CAN (g)	$\bar{M\eta }$	Grafting (G%)	Grafting Efficiency (GE%)	[η_intr_] dL/g
Agar (AA)	-	-	-		175,455	-	-	2.89
A1	1	10	-	0.1	1,011,263	709.0	70.9	10.2
A2	1	10	-	0.2	1,840,781	982.0	98.2	15.7
A3	1	10	-	0.3	1,094,816	764.0	76.4	10.8
A4	1	5	-	0.2	461,687	169.6	33.9	5.8
A5	1	15	-	0.2	1,209,053	779.7	51.9	11.6
M1	1	-	10	0.1	889,499	638.3	63.8	9.3
M2	1	-	10	0.2	1,711,811	966.6	96.6	14.9
M3	1	-	10	0.3	943,076	663.5	66.3	9.7
M4	1	-	5	0.2	418,065	234.2	46.8	5.4
M5	1	-	15	0.2	997,519	668.6	44.5	10.1

**Table 2:** Thermal behavior data.

Sample	Decomposition Stage	Temperature Range (°C) ^a^	DTG Peak (°C) ^b^	Mass Loss ^a^, %	Residue, %
AA	1	~44–130	85	3	97
	2	~225–450	305	56	41
AA-g-AcA (A4)	1	~115–250	230	10	90
	2	~250–320	290	14	73
	3	~320–520	410	44	29
AA-g-McA (M4)	1	~40–125	75	5	95
	2	~130–200	160	13	82
	3	~200–250	230	9	73
	4	~300–520	400	44	29

^a^—From TG. ^b^—From DTG.

**Table 3:** Formulation parameters and characterization of the AA-g-AcA and AA-g-McA nanogels, (EE%—encapsulation efficiency, %).

Polymer	Drug/Polymer Ratio (*w*/*w*)	GA (mL)	EE%
AA-g-AcA	1/1	2	20.81 ± 2.80
	1/1	3	32.66 ± 1.52
	1/1	4	39.12 ± 2.72
AA-g-AcA	1/2	2	35.25 ± 1.42
	1/2	3	56.86 ± 0.03
	1/2	4	62.44 ± 2.40
AA-g-AcA	1/3	2	53.33 ± 2.17
	1/3	3	72.90 ± 1.69
	1/3	4	78.42 ± 1.19
AA-g-McA	1/1	2	19.20 ± 3.20
	1/1	3	30.30 ± 2.15
	1/1	4	40.25 ± 1.35
AA-g-McA	1/2	2	33.15 ± 2.25
	1/2	3	55.60 ± 1.14
	1/2	4	60.98 ± 2.05
AA-g-McA	1/3	2	54.54 ± 2.65
	1/3	3	74.20 ± 0.54
	1/3	4	79.12 ± 2.16

**Table 4:** Average size, polydispersity index (PDI), and zeta potential results from DLS analysis, as well as the equilibrium swelling degree (ESD) in different media.

Parameter	ngAA-g-AcA Empty	ngAA-g-AcA/5-FU	ngAA-g-McA Empty	ngAA-g-McA/5-FU
Size, nm	124.7 ± 7.3	250.0 ± 8.4	105.7 ± 3.3	211.8 ± 4.2
PDI	0.38	0.27	0.39	0.28
Zeta potential, mV	−20.1 ± 3.7	−33.6 ± 3.6	−18.8 ± 2.4	−31.8 ± 3.1
ESD %, pH 5.0	168.2 ± 7.2	-	124.7 ± 6.5	-
ESD %, pH 7.4	465.5 ± 3.8	-	354.9 ± 4.9	-

## Data Availability

The original contributions presented in the study are included in the article, further inquiries can be directed to the corresponding authors.
